# Stiffening symphony of aging: Biophysical changes in senescent osteocytes

**DOI:** 10.1111/acel.14421

**Published:** 2024-11-24

**Authors:** Maryam Tilton, Megan Weivoda, Maria Astudillo Potes, Anne Gingery, Alan Y. Liu, Tamara Tchkonia, Lichun Lu, James L. Kirkland

**Affiliations:** ^1^ Walker Department of Mechanical Engineering The University of Texas at Austin Austin Texas USA; ^2^ Department of Hematology Mayo Clinic Rochester Minnesota USA; ^3^ Department of Biochemistry and Molecular Biology Mayo Clinic Rochester Minnesota USA; ^4^ Department of Physiology and Biomedical Engineering Mayo Clinic Rochester Minnesota USA; ^5^ Department of Orthopedic Surgery Mayo Clinic Rochester Minnesota USA; ^6^ Optics11 Life Inc. Boston Massachusetts USA; ^7^ Robert and Arlene Kogod Center on Aging Mayo Clinic Rochester Minnesota USA

**Keywords:** cellular senescence, cytoskeleton mechanics, osteocyte mechanobiology, sub cellular structure

## Abstract

Senescent osteocytes are key contributors to age‐related bone loss and fragility; however, the impact of mechanobiological changes in these cells remains poorly understood. This study provides a novel analysis of these changes in primary osteocytes following irradiation‐induced senescence. By integrating subcellular mechanical measurements with gene expression analyses, we identified significant, time‐dependent alterations in the mechanical properties of senescent bone cells. Increases in classical markers such as SA‐β‐Gal activity and *p16* ^*Ink4a*^ expression levels confirmed the senescence status post‐irradiation. Our key findings include a time‐dependent increase in cytoskeletal Young's modulus and altered viscoelastic properties of the plasma membrane, affecting the contractility of primary osteocytes. Additionally, we observed a significant increase in Sclerostin (*Sost*) expression 21 days post‐irradiation. These biophysical changes may impair osteocyte mechanosensation and mechanotransduction, contributing to bone fragility. This is the first study to time‐map senescence‐associated mechanical changes in the osteocyte cytoskeleton. Our findings highlight the potential of biophysical markers as indicators of cellular senescence, providing more specificity than traditional, variable biomolecular markers. We believe these results may support biomechanical stimulation as a potential therapeutic strategy to rejuvenate aging osteocytes and enhance bone health.

## Graphical abstract

This study presents a novel analysis of mechanobiological changes in primary osteocytes undergoing irradiation‐induced senescence, focusing on biophysical markers such as cytoskeletal stiffness and membrane viscoelasticity. By integrating subcellular mechanical measurements and gene expression analyses, this work identifies unique biophysical phenotypes of senescent osteocytes, revealing critical alterations in cytoskeletal integrity that may compromise their function and contribute to age‐related changes in bone health.

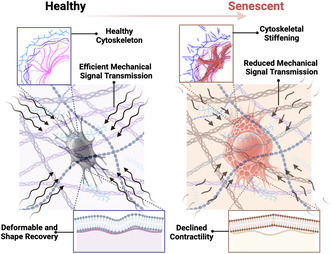

**Abbreviations**
AFM: Atomic Force Microscopy
CTRL: Control
ECM: Extracellular Matrix
FBS: Fetal Bovine Serum
IF: Immunofluorescence
Mepe: Matrix Extracellular Phosphoglycoprotein
Mmp9: Matrix Metalloproteinase 9 p16Ink4a
p21Cip1: Cyclin‐Dependent Kinase Inhibitor 1A (CDKN1A)
SASP: Senescence‐Associated Secretory Phenotype
SA‐β‐Gal: Senescence‐Associated β‐Galactosidase
Sost: Sclerostin
TFM: Traction Force Microscopy; WT, Wild Type

## 1 INTRODUCTION, RESULTS, AND DISCUSSION

Cellular senescence, a hallmark of aging, is linked to a wide range of disorders and diseases, including neurodegenerative conditions, cancer, and degenerative musculoskeletal diseases such as bone loss (Farr & Khosla, 2019). In the United States, over 43 million individuals aged 50 and older have low bone mass, placing them at high risk for osteoporosis and fragility fractures (Sarafrazi et al., 2021). The prevalence of bone loss is notably higher in women compared to men (Sarafrazi et al., 2021). With the global aging population, the economic burden of age‐related fragility fractures is projected to exceed $25 billion by 2025 (Singer et al., 2023). This underscores the critical need to understand the mechanobiological mechanisms underlying age‐related bone loss. Senescent cells (SnCs), including senescent osteocytes, accumulate with age in various tissues, including bone (Farr et al., 2020, 2023). These cells exhibit a senescence‐associated secretory phenotype (SASP), characterized by the secretion of pro‐inflammatory cytokines, chemokines, growth factors, proteases, and other factors, which can damage local and distant tissues and promote chronic inflammation (Farr et al., 2020, 2023).

Osteocytes, once thought to be mere “passive placeholders” in mineralized bone, have emerged as crucial multifunctional cells (Bonewald, 2011; Qin et al., 2020). These “superstars” (Bonewald, 2011) play several essential roles: they are master regulators of bone homeostasis, endocrine cells that regulate phosphate metabolism in organs such as the kidney and parathyroid, and most importantly, they act as strain gauges, regulating bone mechanosensation and mechanotransduction (Bonewald, 2011; Qin et al., 2020). The cytoskeleton of osteocytes, composed of actin microfilaments, microtubules, and intermediate filaments, is crucial for their mechanosensing capabilities (Bonewald, 2011; Qin et al., 2020). Mechanical stimulation induces cytoskeletal rearrangements and stress fiber formation, which are essential for the proper function of osteocytes. However, with aging, the structure and mechanics of the cytoskeleton are altered, impairing osteocyte function and diminishing their mechanosensory properties (Hemmatian et al., 2017). This dysfunction is exacerbated by the accumulation of SnCs and their SASP, both locally and systemically (Farr et al., 2023). These cells express markers of senescence, such as the cyclin‐dependent kinase inhibitors *p16* ^*Ink4a*^ (encoded by *Cdkn2a*) and *p21* ^*Cip1*^ (encoded by *Cdkn1a*), which are part of the stress response program that drives cellular aging (Farr et al., 2023).

Current research has primarily relied on SA‐β‐Gal activity, expression of senescence and senescence‐associated genomic markers, chromosomal changes, as well as a combination of SA‐β‐Gal and flow cytometry to identify senescence status (Farr et al., 2016). However, these biomarkers are highly variable across different cell types and tissues (Chaib et al., 2022; Saul et al., 2022). Additionally, there is evidence of sex‐dependent variations in senescence‐associated factors (Farr et al., 2016; Saul et al., 2022). Therefore, it is crucial to explore alternative biophysical markers that provide unique and reliable fingerprints of aging. Understanding changes in cytoskeletal mechanics could lay the foundation for future research aimed at reversing or rejuvenating aging cells through biomechanical stimulation. Moreover, establishing such biophysical markers could complement the growing set of genetic markers in this research space (Saul et al., 2022), offering more consistent and robust tools for identifying senescence and reducing reliance on the highly variable biomolecular markers currently in use.

Here, we present studies of novel biophysical markers of senescent primary osteocytes, focusing on changes in the mechanical properties of the plasma membrane and cytoskeleton following irradiation‐induced senescence. By characterizing these biophysical alterations, we aim to establish reliable markers that can enhance understanding of cellular senescence and support the development of therapeutic strategies to counteract the effects of aging on bone health and potentially other tissues and organs.

**FIGURE 1:**
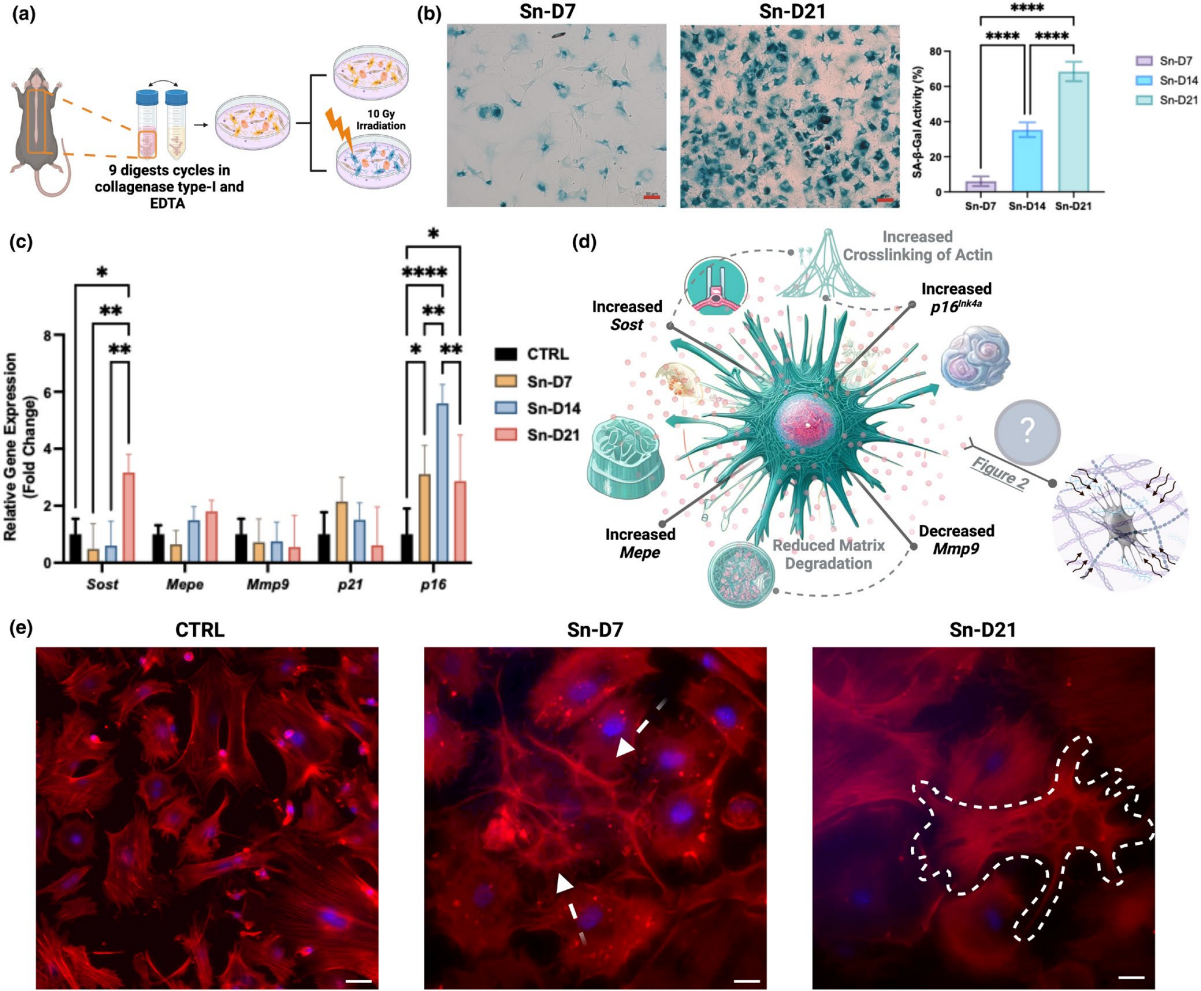
Overview of the study and gene expression changes in senescent primary osteocytes. (a) Schematic representation of primary osteocyte isolation, culture, and induction of senescence by irradiation. (b) Bright field microscopy of irradiated primary cell culture showing increased in SA‐β‐Gal activity with peaking to over 70% by day 21 [scale bar = 50 μm]. (c, d) Normalized mRNA expression levels of key osteocyte and senescence‐associated genes. Increases in Sost and Mepe, along with a decrease in Mmp9, were observed in senescent osteocytes compared to non‐senescent control cells. Elevated p16 expression, along with increased SA‐β‐Gal activity (b), confirmed the senescence status. As illustrated in representative immunofluorescence staining images (F‐actin/DAPI) [scale bar = 25 μm], (e) changes in cell size, dendritic network (arrows in Sn‐D7), and morphology (dashed boundary in Sn‐D21) were observed. We have provided additional IF images in Appendix S1. These micrographs, from different regions of the CTRL and Sn‐D21 cultures, allow for better observation of the morphological changes due to senescence condition across different regions. Data are presented as means ± SD, with statistical significance denoted by *p < 0.05, **p < 0.01, ***p < 0.001, and ****p < 0.0001.

As detailed in Appendix S1, primary osteocytes were isolated from vertebrae of 10 young (4 months, female) C57BL/6 WT mice using an established protocol from Linda Bonewald's group (Stern et al., 2012), a method that our team has extensively utilized in previous publications (Figure 1a) (Farr et al., 2016, 2023). The significant amount of trabecular bone in the vertebrae provides a larger surface area and easier accessibility, which are critical for efficient osteocyte extraction. A mixture of 5% fetal bovine serum (FBS) and 5% calf serum was used in the α‐MEM growth medium to optimize the environment for osteocyte viability and maintenance. This approach balances growth support with the preservation of the osteocyte phenotype, minimizing the risk of unwanted differentiation and ensuring that the cells retain their specific characteristics throughout the experimental procedures. We used low‐dose irradiation (10 Gy) as a pro‐senescence stressor to induce senescence in primary osteocyte cultures in vitro. All culture plates were coated with rat tail type I collagen (0.15 mg/mL). Maintaining three wells per study group for every experiment, we investigated changes in single‐cell mechanics at different time points post‐irradiation (D7, D14, and D21) and compared them to a non‐senescent control group (CTRL).

We used optical fiber‐based interferometry nanoindentation (Pavone, Optics11Life) with an indenter tip size of R = 3 μm and stiffness of 0.019 N/m on live cells, enabling time‐lapsed single‐cell analyses of senescence‐associated changes in cytoskeletal modulus and membrane viscoelasticity. We included a minimum of 30 cells per well in our single‐cell nanoindentation experiments, ensuring a minimum of 90 cells per study group per time point were tested, distributed across multiple wells. Additionally, to account for potential time‐dependent changes in membrane properties, we included time‐matched controls for each experimental point (days 7, 14, and 21). The control data were combined across time points due to the lack of significant changes, ensuring that observed differences were attributable to senescence rather than time‐related artifacts.

The senescence status of the cultures was determined by SA‐β‐Gal activity (Figure 1b). Real‐time quantitative polymerase chain reaction (RT‐qPCR) was performed to measure changes in the expression of senescence markers (e.g., *p16* ^*Ink4a*^ and *p21* ^*Cip1*^) (Figure 1c). Additionally, key genomic markers associated with osteocytes, the Wnt/β‐catenin signaling pathway, and mineralization, such as sclerostin (*Sost*), matrix extracellular phosphoglycoprotein (*Mepe*), and matrix metalloproteinases (*Mmp9*), were measured using RT‐qPCR (Figure 1c,d).

**FIGURE 2:**
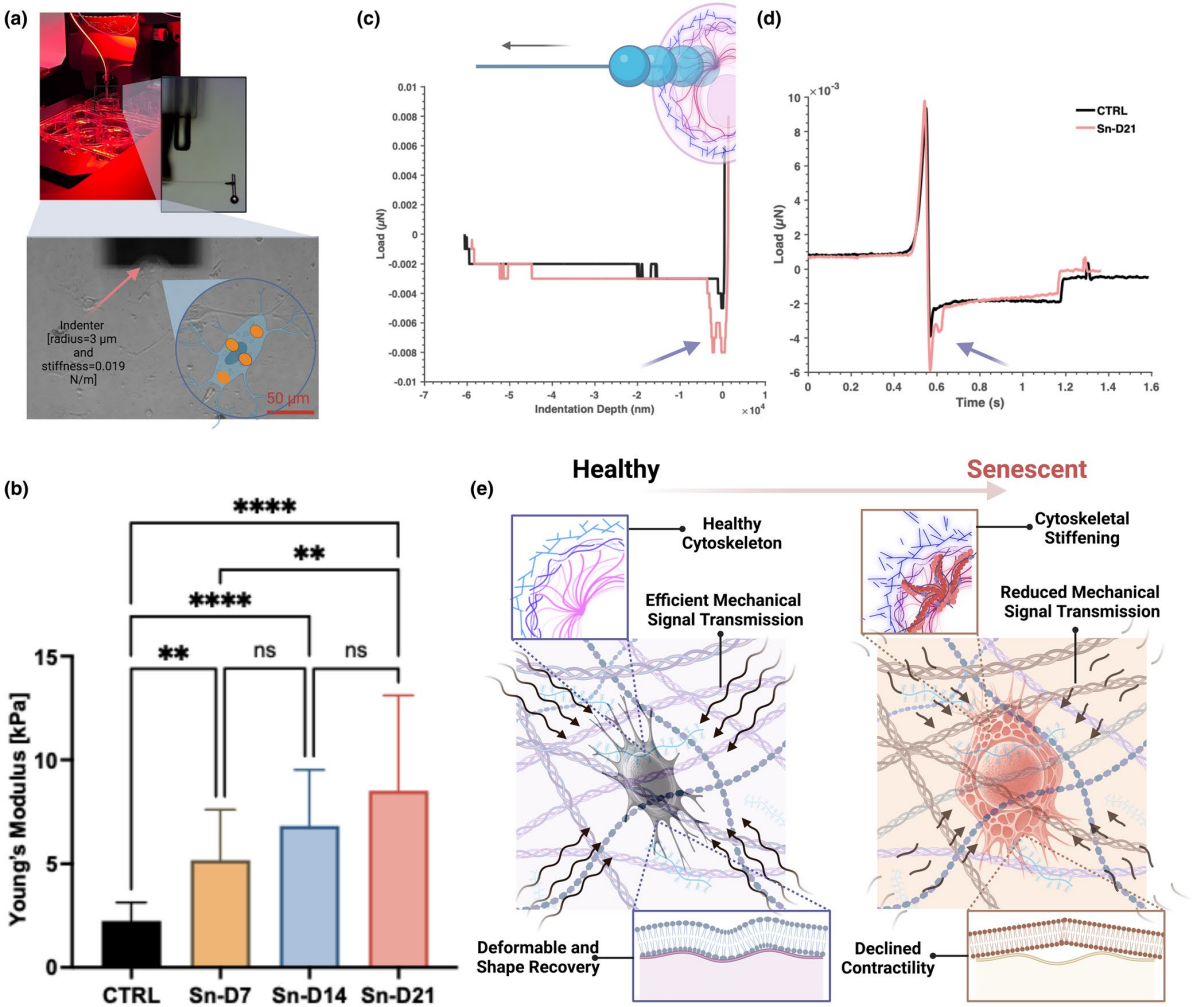
Mechanobiological Changes in Senescent Osteocytes. (a) Representative images of our mechanobiology experimental setup (b) Mechanical measurements showing increased Young's modulus and potentially altered viscoelastic properties in senescent osteocytes. A minimum of *N* = 90 single cells per study group (i.e., CTRL, Sn‐D7, Sn‐D14, and Sn‐D21) were mechanically tested at every time point. Young's modulus was obtained using the Hertzian contact model with Poisson's ratio ν=0.5. (c) Mean unloading portions of indentation curves and (d) load‐time curves, demonstrating significantly higher minimum adhesion force and delayed recovery in senescent cells (*p* = 0.0084). In plot (c), all indentation data from control and Sn‐D21 were used, while plot (d) includes experimental data from cells exhibiting osteocyte‐like morphology. (e) Schematic drawing connecting these findings to the impaired mechanosensation and mechanotransduction capabilities of senescent osteocytes. These changes contribute to age‐related bone loss and fragility. Data are presented as means ± SD, with statistical significance denoted by *p < 0.05, **p < 0.01, ***p < 0.001, and ****p < 0.0001.

As seen in Figure 2, using the Hertzian contact mechanics model with our load‐indentation data (Qian & Zhao, 2018), our findings indicated a significant increase in Young's modulus of cells on D7, D14, and D21 post‐irradiation compared to the CTRL group. Although the changes in modulus from D7 to D14 and D14 to D21 were not statistically significant, an increasing trend was observed, with a statistically significant increase at D21 compared to D7 (Figure 2b). Dissecting the unloading portion of indentation curves (Figure 2c) revealed a smoother recovery phase with a low adhesion force (range: 0.002–0.005 μN) in the CTRL groups, indicating that healthy primary cells exhibit predominantly elastic behavior. In contrast, senescent cells exhibited higher minimum adhesion force (range: 0.006–0.01 μN) with a noticeable secondary peak or “bump” before returning to the baseline load. Analyzing the unloading portion of load‐time graphs (Figure 2d), healthy cells showed smooth and rapid recovery to the baseline load, likely due to better stress relaxation capabilities. However, in senescent cells, the fluctuations (i.e., secondary peak) after the minimum adhesion force and the extended recovery time suggest heterogeneous viscoelastic properties and delayed mechanical recovery, indicating altered cytoskeletal architecture and function, a hallmark of senescence. Specifically, the wider interval between the minimum adhesion force and the return to the baseline load in senescent cells demonstrates the prolonged interaction between the indenter and the cells due to altered viscoelastic properties. These observations mark distinct biophysical fingerprints of cellular senescence. A previous study (Ghosh et al., 2020) on senescent mesenchymal stem cells showed that irradiation‐induced senescence led to cytoskeletal reorganization through formation of stress fibers, increased crosslinking of actin filaments, and a denser actin network. The cytoskeletal stiffening process increases the cells' resistance to deformation and slows their shape recovery.

These new experimental outcomes provide a foundational understanding of the mechanisms by which the cytoskeletal integrity of osteocytes alters as they become senescent, potentially compromising their ability to sense and respond to mechanical stimuli (see Figure 2e). Probing these biophysical changes alongside gene expression variations (see Figure 1c) helped us to decipher biophysical implications of biomolecular changes in cellular senescence. *Sost*, an inhibitor of the Wnt/β‐catenin signaling pathway, was notably upregulated at day 21 (Sn‐D21). This may suggest that *Sost* might play a crucial role in cytoskeletal reorganization and the resultant increased stiffness through an indirect interaction with the Wnt/β‐catenin pathway (Canalis, 2013; Wu et al., 2017). It has been suggested that a divergent canonical Wnt pathway regulates the microtubule cytoskeleton to promote cell movement, indicating that Wnt signaling influences the cytoskeletal dynamics (Chen et al., 2016). According to a previous study (Cho et al., 2012), Wnt3a directly stimulates *Mepe* transcription via β‐catenin and Lef‐1 and indirectly through an autocrine BMP‐2 loop. These pathways play crucial roles in bone homeostasis and remodeling. Here, a slight increase in expression level of *Mepe* observed on days 14 and 21 post‐irradiation (Figure 1d,e) likely reflects adaptive response of primary osteocytes to irradiation, potentially contributing to cytoskeletal stiffening. Additionally, the decreasing expression trend of *Mmp9* from CTRL to Sn‐D21 (see Figure 1d) could be linked to reduced ECM degradation. This reduction may contribute to a more rigid cytoskeletal structure in senescent cells (Chen et al., 2016). Thus, while *Mepe* and *Mmp9* are two of the primary markers associated with mineralization and ECM remodeling, their altered expression in senescent cells underscores broader implications for cytoskeletal organization and mechanical properties.

The significant increase in *p16* ^*Ink4a*^ expression level, a potent cyclin‐dependent kinase inhibitor, has been previously linked to bone loss and age‐related pathologies (Farr et al., 2020, 2023). Elevated p16^Ink4a^ levels are associated with the accumulation of senescent cells, which contribute to impaired tissue function and increased fracture risk in osteoporosis (Farr et al., 2020, 2023). The causal role of p16^high^ cells has been implicated in various pathological conditions, including osteoporosis (Farr et al., 2020, 2023). Our study primarily aimed to identify biophysical phenotypes in senescent osteocytes, with RT‐qPCR conducted to confirm senescence status and assess changes in key biomarkers. In Figure 1c, the observed inverse trends in *Sost*, *p21* ^*Cip1*^, and *p16* ^*Ink4a*^ expression between Day 21 and earlier time points may reflect dynamic regulatory mechanisms during senescence progression, as well as the impact of culture heterogeneity. While RNA‐level data offers valuable insights, we acknowledge the importance of protein‐level validation. However, such analyses were beyond the scope of this study. Future research should incorporate these analyses to further strengthen our findings and explore the broader implications of these gene expression changes. Overall, these observations align with previous studies (Farr et al., 2020, 2023) that have shown senescent osteocytes exhibit increased *p16* ^*Ink4a*^ expression with age, contributing to reduced bone remodeling capacity.

In vivo, osteocytes are confined within lacunae and surrounded by a mineralized ECM, which likely restricts the cell size increases, and cytoplasmic spreading observed in our 2D in vitro experiments. The cytoskeletal disorganization we observed at day 21, particularly in F‐actin distribution, likely resulted from oxidative stress induced by irradiation (Milzani et al., 1997). This disorganization may disrupt both mechanical responses and intracellular signaling, potentially compromising bone integrity (Chowdhury et al., 2021; Gould et al., 2021; Hemmatian et al., 2018; Kim et al., 2022; Tiede‐Lewis & Dallas, 2019). However, the physical constraints imposed by the lacunocanalicular system and the ECM in vivo would likely lead to different mechanical consequences than those observed in vitro. It is worth noting that several previous studies have reported enlargement of cell soma or cytoplasm, both in vitro and in vivo, as a hallmark of cellular senescence (Biran et al., 2017; Lanz et al., 2022; Ogrodnik et al., 2021, 2024). However, determining such phenomena specifically in bone tissue in vivo remains challenging. Thus, future studies employing more advanced characterization and imaging techniques are required to validate this senescence‐associated morphological phenotype in bone tissue (Ogrodnik et al., 2024). Additionally, age‐related changes in lacuna morphology, such as reduced size and increased sphericity (Hemmatian et al., 2018; Heveran et al., 2018; Tiede‐Lewis & Dallas, 2019; Zhang et al., 2019), may further alter osteocyte mechanobiology, affecting their ability to transmit mechanical signals within the bone matrix. The enlargement and mechanical stiffening observed in our 2D model, therefore, likely represent exaggerated responses due to the absence of these constraints, highlighting the limitations of 2D culture systems in replicating the complex in vivo environment.

Nonetheless, our 2D model allows for direct characterization of single‐cell micromechanical properties, which are critical for understanding the senescence‐induced functional decline of osteocytes. Such precise measurements are challenging to achieve in vivo or in 3D models, where the biomechanical focus often shifts to the properties of the surrounding ECM rather than the cells themselves. To address these limitations, future studies will integrate AI‐driven approaches to predict morphological phenotypes from image‐based data and employ digital twin methods for physics‐based simulations of osteocyte mechanics. Integrating these advanced tools with novel 3D co‐culture models will facilitate the translation of our in vitro findings to more physiologically relevant conditions. Such an approach will enable more accurate investigation of cell–cell and cell‐ECM interactions under both physiological and pathological conditions, offering deeper insights into osteocyte senescence and its impact on bone health.

To date, there are only two published reports that have investigated changes in the mechanical properties of senescent cells at the single‐cell level, both focusing on fibroblasts and skin as their model systems (Brauer et al., 2023; Rebehn et al., 2023). Therefore, the present study is pioneering in exploring senescence‐induced changes in the mechanical properties of primary osteocytes, which are the most abundant mechanosensory cells in the human body. Previous studies (Brauer et al., 2023; Rebehn et al., 2023) have used either traction force microscopy (TFM) or atomic force microscopy (AFM). While these methods have contributed significantly to our understanding, they have limitations that our mechanobiology experimental setup overcomes. TFM's reliance on the mechanics of the PDMS substrate for cellular force inference introduces complexities, while AFM, though direct in force application, cannot achieve the high specificity in indenting as well as our method. The use of a sharp, diamond‐shaped indenter in AFM can also affect outcomes in single‐cell studies. Moreover, non‐invasive and real‐time monitoring techniques such as those used in this study can be adapted for real‐time assessment of cell mechanics in living tissues. These methods allow biophysical markers to be assessed in live cells without the need for fixation or labeling, preserving the natural state of the cells and enabling longitudinal studies of senescence and aging. Overall, our approach enhances the ability to monitor biophysical markers of single cells over time, providing valuable insights into the dynamics of cellular aging.

In this study, we focused on identifying senescence‐associated biophysical markers specifically in osteocytes, as these cells play a crucial role in bone health and are directly implicated in age‐related bone disorders. While cross‐cell‐type comparisons, such as with skin fibroblasts, could provide further insights into the generalizability of these findings, our current research is specifically tailored to understanding the biophysical changes within the bone microenvironment. Future studies will be needed to determine whether these biophysical changes are unique to osteocytes or represent a broader characteristic of cellular senescence across different tissues.

In summary, our study identifies novel biophysical markers of senescent bone cells, particularly alterations in their cytoskeletal mechanics, such as changes in stiffness and structural integrity. Although our current findings do not directly measure mechanosensation and mechanotransduction, they provide a crucial foundation for understanding how cellular senescence may compromise the mechanical integrity of bone cells. These insights are essential as they set the stage for our future investigations that will directly assess the mechanosensory and mechanotransduction functions in bone cells, particularly in response to mechanical stimuli within a dynamic microenvironment, through controlled accumulation of senescent cells. The correlation between gene expression changes and mechanical properties underscores the importance of using such biophysical markers in conjunction with highly variable genomic markers to provide more reliable indicators of cellular senescence. Additionally, our new findings further support the hypothesis that biomechanical stimulation or exercise of cells could be used as a therapeutic approach to counteract the effects of aging on osteocytes. Specifically, by targeting the mechanobiological pathways involved in cytoskeletal stiffening, it may be possible to rejuvenate aging cells and improve bone health in elderly populations. An intriguing question for future research is whether these biophysical fingerprints (i.e., mechanical markers) could provide a better indication of whether the senescence state of a cell is beneficial or detrimental. This understanding could significantly influence how we perceive and manage cellular senescence in osteocytes and potentially other cell types within the musculoskeletal system.

## AUTHOR CONTRIBUTIONS

Maryam Tilton, Megan Weivoda, and James Kirkland designed the study. Maryam Tilton performed the experiments, researched, analyzed, interpreted the data, and wrote the original manuscript. Maryam Tilton, Megan Weivoda, Maria Astudillo Potes, Anne Gingery, Alan Liu, Tamara Tchkonia, Lichun Lu, and James Kirkland provided resources. All authors reviewed and revised the manuscript. All authors read and approved the final manuscript.

## CONFLICT OF INTEREST STATEMENT

None declared.

## Supporting information


Appendix S1.

## Data Availability

The data that support the findings of this study are available on request from the corresponding author.

## References

[acel14421-bib-0001] Biran, A. , Zada, L. , Abou Karam, P. , Vadai, E. , Roitman, L. , Ovadya, Y. , Porat, Z. , & Krizhanovsky, V. (2017). Quantitative identification of senescent cells in aging and disease. Aging Cell, 16(4), 661–671. 10.1111/ACEL.12592 28455874 PMC5506427

[acel14421-bib-0002] Bonewald, L. F. (2011). The amazing osteocyte. Journal of Bone and Mineral Research, 26(2), 229–238. 10.1002/JBMR.320 21254230 PMC3179345

[acel14421-bib-0003] Brauer, E. , Lange, T. , Keller, D. , Görlitz, S. , Cho, S. , Keye, J. , Gossen, M. , Petersen, A. , & Kornak, U. (2023). Dissecting the influence of cellular senescence on cell mechanics and extracellular matrix formation in vitro. Aging Cell, 22(3), e13744. 10.1111/acel.13744 36514868 PMC10014055

[acel14421-bib-0004] Canalis, E. (2013). Wnt signalling in osteoporosis: Mechanisms and novel therapeutic approaches. Nature Reviews Endocrinology, 9(10), 575–583. 10.1038/nrendo.2013.154 23938284

[acel14421-bib-0005] Chaib, S. , Tchkonia, T. , & Kirkland, J. L. (2022). Cellular senescence and senolytics: The path to the clinic. Nature Medicine, 28(8), 1556–1568. 10.1038/s41591-022-01923-y PMC9599677 35953721

[acel14421-bib-0006] Chen, J. , Rajasekaran, M. , Xia, H. , Zhang, X. , Kong, S. N. , Sekar, K. , Seshachalam, V. P. , Deivasigamani, A. , Goh, B. K. P. , Ooi, L. L. , Hong, W. , & Hui, K. M. (2016). The microtubule‐associated protein PRC1 promotes early recurrence of hepatocellular carcinoma in association with the Wnt/β‐catenin signalling pathway. Gut, 65(9), 1522–1534. 10.1136/GUTJNL-2015-310625 26941395 PMC5036256

[acel14421-bib-0007] Cho, Y. D. , Kim, W. J. , Yoon, W. J. , Woo, K. M. , Baek, J. H. , Lee, G. , Kim, G. S. , & Ryoo, H. M. (2012). Wnt3a stimulates Mepe, matrix extracellular phosphoglycoprotein, expression directly by the activation of the canonical Wnt signaling pathway and indirectly through the stimulation of autocrine bmp‐2 expression. Journal of Cellular Physiology, 227(6), 2287–2296. 10.1002/JCP.24038 22213482

[acel14421-bib-0008] Chowdhury, F. , Huang, B. , & Wang, N. (2021). Cytoskeletal prestress: The cellular hallmark in mechanobiology and mechanomedicine. Cytoskeleton, 78(6), 249–276. 10.1002/cm.21658 33754478 PMC8518377

[acel14421-bib-0009] Farr, J. N. , Fraser, D. G. , Wang, H. , Jaehn, K. , Ogrodnik, M. B. , Weivoda, M. M. , Drake, M. T. , Tchkonia, T. , LeBrasseur, N. K. , Kirkland, J. L. , Bonewald, L. F. , Pignolo, R. J. , Monroe, D. G. , & Khosla, S. (2016). Identification of senescent cells in the bone microenvironment. Journal of Bone and Mineral Research, 31(11), 1920–1929. 10.1002/jbmr.2892 27341653 PMC5289710

[acel14421-bib-0010] Farr, J. N. , Kaur, J. , Doolittle, M. L. , Khosla, S. , Farr, J. , & Doolittle, M. (2020). Osteocyte Cellular Senescence. Current Osteoporosis Reports, 18(5), 559–567. 10.1007/s11914-020-00619-x 32794138 PMC7541777

[acel14421-bib-0011] Farr, J. N. , & Khosla, S. (2019). Cellular senescence in bone. Bone, 121, 121–133. 10.1016/j.bone.2019.01.015 30659978 PMC6485943

[acel14421-bib-0012] Farr, J. N. , Saul, D. , Doolittle, M. L. , Kaur, J. , Rowsey, J. L. , Vos, S. J. , Froemming, M. N. , Lagnado, A. B. , Zhu, Y. , Weivoda, M. , Ikeno, Y. , Pignolo, R. J. , Niedernhofer, L. J. , Robbins, P. D. , Jurk, D. , Passos, J. F. , LeBrasseur, N. K. , Tchkonia, T. , Kirkland, J. L. , … Khosla, S. (2023). Local senolysis in aged mice only partially replicates the benefits of systemic senolysis. The Journal of Clinical Investigation, 133(8), 366–374. 10.1172/JCI162519 PMC10104901 36809340

[acel14421-bib-0013] Ghosh, D. , Mejia‐Pena, C. , Quach, N. , Xuan, B. , Lee, A. H. , & Dawson, M. R. (2020). Senescent mesenchymal stem cells remodel extracellular matrix driving breast cancer cells to a more‐invasive phenotype. Journal of Cell Science, 133(2), jcs232470. 10.1242/JCS.232470 31932504 PMC6983709

[acel14421-bib-0014] Gould, N. R. , Torre, O. M. , Leser, J. M. , & Stains, J. P. (2021). The cytoskeleton and connected elements in bone cell mechano‐transduction. Bone, 149, 115971. 10.1016/j.bone.2021.115971 33892173 PMC8217329

[acel14421-bib-0015] Hemmatian, H. , Bakker, A. D. , Klein‐Nulend, J. , & van Lenthe, G. H. (2017). Aging, osteocytes, and Mechanotransduction. Current Osteoporosis Reports, 15(5), 401–411. 10.1007/s11914-017-0402-z 28891009 PMC5599455

[acel14421-bib-0016] Hemmatian, H. , Laurent, M. R. , Bakker, A. D. , Vanderschueren, D. , Klein‐Nulend, J. , & van Lenthe, G. H. (2018). Age‐related changes in female mouse cortical bone microporosity. Bone, 113, 1–8. 10.1016/J.BONE.2018.05.003 29738854

[acel14421-bib-0017] Heveran, C. M. , Rauff, A. , King, K. B. , Carpenter, R. D. , & Ferguson, V. L. (2018). A new open‐source tool for measuring 3D osteocyte lacunar geometries from confocal laser scanning microscopy reveals age‐related changes to lacunar size and shape in cortical mouse bone. Bone, 110, 115–127. 10.1016/J.BONE.2018.01.018 29374550 PMC5878731

[acel14421-bib-0018] Kim, Y. J. , Cho, M. J. , Yu, W. D. , Kim, M. J. , Kim, S. Y. , & Lee, J. H. (2022). Links of cytoskeletal integrity with disease and aging. Cells, 11(18), 2896. 10.3390/cells11182896 36139471 PMC9496670

[acel14421-bib-0019] Lanz, M. C. , Zatulovskiy, E. , Swaffer, M. P. , Zhang, L. , Ilerten, I. , Zhang, S. , You, D. S. , Marinov, G. , McAlpine, P. , Elias, J. E. , & Skotheim, J. M. (2022). Increasing cell size remodels the proteome and promotes senescence. Molecular Cell, 82(17), 3255–3269.e8. 10.1016/J.MOLCEL.2022.07.017 35987199 PMC9444988

[acel14421-bib-0020] Milzani, A. , DalleDonne, I. , & Colombo, R. (1997). Prolonged oxidative stress on actin. Archives of Biochemistry and Biophysics, 339(2), 267–274. 10.1006/abbi.1996.9847 9056258

[acel14421-bib-0021] Ogrodnik, M. , Carlos Acosta, J. , Adams, P. D. , d'Adda di Fagagna, F. , Baker, D. J. , Bishop, C. L. , Chandra, T. , Collado, M. , Gil, J. , Gorgoulis, V. , Gruber, F. , Hara, E. , Jansen‐Dürr, P. , Jurk, D. , Khosla, S. , Kirkland, J. L. , Krizhanovsky, V. , Minamino, T. , Niedernhofer, L. J. , … Demaria, M. (2024). Guidelines for minimal information on cellular senescence experimentation in vivo. Cell, 187(16), 4150–4175. 10.1016/J.CELL.2024.05.059 39121846 PMC11790242

[acel14421-bib-0022] Ogrodnik, M. , Evans, S. A. , Fielder, E. , Victorelli, S. , Kruger, P. , Salmonowicz, H. , Weigand, B. M. , Patel, A. D. , Pirtskhalava, T. , Inman, C. L. , Johnson, K. O. , Dickinson, S. L. , Rocha, A. , Schafer, M. J. , Zhu, Y. , Allison, D. B. , von Zglinicki, T. , LeBrasseur, N. K. , Tchkonia, T. , … Jurk, D. (2021). Whole‐body senescent cell clearance alleviates age‐related brain inflammation and cognitive impairment in mice. Aging Cell, 20(2), e13296. 10.1111/ACEL.13296 33470505 PMC7884042

[acel14421-bib-0023] Qian, L. , & Zhao, H. (2018). Nanoindentation of soft biological materials. Micromachines, 9(12), 654. 10.3390/MI9120654 30544918 PMC6316095

[acel14421-bib-0024] Qin, L. , Liu, W. , Cao, H. , & Xiao, G. (2020). Molecular mechanosensors in osteocytes. Bone Research, 8, 23. 10.1038/s41413-020-0099-y 32550039 PMC7280204

[acel14421-bib-0025] Rebehn, L. , Khalaji, S. , KleinJan, F. , Kleemann, A. , Port, F. , Paul, P. , Huster, C. , Nolte, U. , Singh, K. , Kwapich, L. , Pfeil, J. , Pula, T. , Fischer‐Posovszky, P. , Scharffetter‐Kochanek, K. , & Gottschalk, K. E. (2023). The weakness of senescent dermal fibroblasts. Proceedings of the National Academy of Sciences of the United States of America, 120(34), e2301880120. 37579160 10.1073/pnas.2301880120 PMC10450655

[acel14421-bib-0026] Sarafrazi, N. , Wambogo, E. A. , & Shepherd, J. A. (2021). Osteoporosis or low bone mass in older adults: United States, 2017–2018. NCHS Data Brief, 405, 1–8. 34029181

[acel14421-bib-0027] Saul, D. , Kosinsky, R. L. , Atkinson, E. J. , Doolittle, M. L. , Zhang, X. , LeBrasseur, N. , Pignolo, R. J. , Robbins, P. D. , Niedernhofer, L. J. , Ikeno, Y. , Jurk, D. , Passos, J. F. , Hickson, L. J. , Xue, A. , Monroe, D. G. , Tchkonia, T. , Kirkland, J. L. , Farr, J. N. , & Khosla, S. (2022). A new gene set identifies senescent cells and predicts senescence‐associated pathways across tissues. Nature Communications, 13(1), 1–15. 10.1038/s41467-022-32552-1 PMC9381717 35974106

[acel14421-bib-0028] Singer, A. , Mcclung, M. R. , Tran, O. , Morrow, C. D. , Goldstein, S. , Kagan, R. , McDermott, M. , & Yehoshua, A. (2023). Treatment rates and healthcare costs of patients with fragility fracture by site of care: A real‐world data analysis. Archives of Osteoporosis, 18(1), 42. 10.1007/s11657-023-01229-7 36905559 PMC10008255

[acel14421-bib-0029] Stern, A. R. , Stern, M. M. , van Dyke, M. E. , Jähn, K. , Prideaux, M. , & Bonewald, L. F. (2012). Isolation and culture of primary osteocytes from the long bones of skeletally mature and aged mice. Biotechniques, 52(6), 361–373. 10.2144/0000113876 22668415 PMC3612989

[acel14421-bib-0030] Tiede‐Lewis, L. A. M. , & Dallas, S. L. (2019). Changes in the osteocyte lacunocanalicular network with aging. Bone, 122, 101–113. 10.1016/J.BONE.2019.01.025 30743014 PMC6638547

[acel14421-bib-0031] Wu, T. , Wang, L. N. , Tang, D. R. , & Sun, F. Y. (2017). SOST silencing promotes proliferation and invasion and reduces apoptosis of retinoblastoma cells by activating Wnt/β‐catenin signaling pathway. Gene Therapy, 24(7), 399–407. 10.1038/gt.2017.31 28485721

[acel14421-bib-0032] Zhang, C. , Bakker, A. D. , Klein‐Nulend, J. , & Bravenboer, N. (2019). Studies on osteocytes in their 3D native matrix versus 2D in vitro models. Current Osteoporosis Reports, 17(4), 207–216. 10.1007/S11914-019-00521-1/FIGURES/2 31240566 PMC6647862

