# Supplementary material for: Stiffening symphony of aging: Biophysical changes in senescent osteocytes
Source: Aging Cell. 2024 Nov 24;23(12):e14421. doi: 10.1111/acel.14421 (PMC11634739; doi:10.1111/acel.14421)
Supplement: Supplementary file 1 — Appendix S1. [file ACEL-23-e14421-s001.docx]

**Stiffening Symphony of Aging: Biophysical Changes in Senescent Osteocytes**

Maryam Tilton^1^*, Megan Weivoda^2,3^, Maria Astudillo Potes^4,5^, Anne Gingery^3,4,5,^, Alan Y. Liu^6^, Tamara Tchkonia^4,7^, Lichun Lu^4,5^, James L. Kirkland^4,7^

^1^ Walker Department of Mechanical Engineering, The University of Texas at Austin, Austin, Texas, USA

^2^ Department of Hematology, Mayo Clinic, Rochester, Minnesota, USA

^3^ Department of Biochemistry and Molecular Biology, Mayo Clinic, Rochester, Minnesota, USA

^4^ Department of Physiology and Biomedical Engineering, Mayo Clinic, Rochester, Minnesota, USA

^5^ Department of Orthopedic Surgery, Mayo Clinic, Rochester, Minnesota, USA

^6^ Optics11 Life Inc., Boston, Massachusetts, USA

^7^ Robert and Arlene Kogod Center on Aging, Mayo Clinic, Rochester, Minnesota, USA

*Corresponding Author:

Maryam Tilton, Ph.D.

Walker Department of Mechanical Engineering, The University of Texas at Austin, Austin, Texas, USA

Email: [maryam.tilton@austin.utexas.edu](mailto:maryam.tilton@austin.utexas.edu)

**Supplementary Methods**

**Primary osteocyte isolation, culture, and senescence induction:** Following the previously established protocol,^1–3^ primary osteocytes were isolated from vertebrae of C57BL/6 WT mice (N=10; female). A highly enriched population of osteocytes were collected from digestion cycles 7 to 9, centrifuged, and seeded on 0.15 mg/mL rat tail type I collagen-coated plates (Fisher Scientific). ^4^ The culture medium comprised ⍺-MEM (Sigma), enriched with L-glutamine, nucleosides, 5% heat-inactivated fetal bovine serum, and 5% calf serum (both from Sigma). After two passages to ensure cell purity and viability, the cells were cultured in type I collagen-coated 6-well plates. A subset of these cultures was exposed to 10 Gy of cesium irradiation (CellRad, Precision X-Ray) to induce senescence *in vitro*. ^1,2^ Each experimental condition was replicated across three wells.

To account for potential time-dependent changes in membrane properties and biophysical markers of the primary bone cells, we implemented a comprehensive control strategy throughout our study. Four 6-well plates were allocated for the control healthy condition (CTRL). For each experimental time point (days 7, 14, and 21), one 6-well plate was analyzed in parallel with the corresponding senescent conditions. From each 6-well plate, 3 wells were dedicated to single-cell nanoindentation and immunofluorescence (IF) assays, while the remaining 3 wells were utilized for RNA extraction and RT-qPCR analysis.

In addition, a fourth control plate was maintained throughout the entire study duration, with day 21 as the endpoint. This plate was used exclusively for single-cell nanoindentation measurements to assess any time-dependent changes in both biophysical and biomolecular markers under healthy conditions. By doing so, we ensured that any observed differences between control and senescent cells could be confidently attributed to senescence-induced changes rather than time-related artifacts.

Given that no statistically significant changes in the cytoskeletal Young’s modulus were observed between days 7 and 21 in the control condition, the data from the control cultures across all time points were combined for analysis.

For the irradiated senescent conditions, a similar approach was applied, where an irradiated 6-well plate was maintained and tested for cytoskeletal mechanics at each time point (days 7, 14, and 21). The results from these time points were collected in parallel with the dedicated 6-well plates for each senescent study group (i.e., Sn-D7, Sn-D14, and Sn-D21), allowing us to effectively monitor the progression of senescence and its impact on cellular mechanics.

**Senescence associated β-Galactosidase (SA-β-Gal) assay:** Following the previously established protocol,^5^ cellular senescence was assessed by measuring SA-β-Gal activity, a reliable marker for senescent cells, using the CellSignaling SA-β-Gal staining kit, as per the manufacturer's instructions. The SA-β-Gal assay is currently the most widely used method for detecting senescence at the single-cell level due to its convenience and effectiveness. For quantitative analysis, ten randomly selected fields of view (both central and peripheral) were captured for each sample from each experimental group using brightfield inverted microscopy in a blinded manner. To quantify SA-β-Gal activity (%), an intensity-based analysis was conducted using ImageJ software. Brightfield microscopy images were converted to 8-bit grayscale to focus on the intensity of staining. A consistent threshold was applied to distinguish positive SA-β-Gal staining from the background, determined by the histogram of pixel intensity. The 'Analyze Particles' feature in ImageJ quantified the area stained relative to the total field area, providing a measure of SA-β-Gal activity as a percentage of the senescent cells.

**Real-Time quantitative polymerase chain reaction (RT-qPCR):** For RT-qPCR, biomarker tracking was systematically performed at various culture time-points (days 7, 14, and 21), utilizing a methodology extensively described in previous literature. ^1–4^ RNA isolation was achieved using Trizol reagent coupled with mechanical homogenization (i.e., ceramic bead mill tubes), followed by purification with the GeneJet RNA Purification Kit (Thermo Fisher). The RNA concentration was determined using the NanoDrop One spectrophotometer (Thermo Fisher). Subsequently, the purified RNA from each study group and time point was reverse transcribed to cDNA using the SuperScript IV VILO Master Mix (Thermo Fisher). In this pilot study, we focused on evaluating the expression levels of key biomarkers relevant to primary osteocyte function and senescence. Specifically, the expression of osteocyte-specific markers such as Matrix Extracellular Phosphoglycoprotein (MEPE) and Sclerostin (SOST), along with well-validated chronic senescence effectors in bone tissue like p16^Ink4a^ and p21, and Matrix Metalloproteinases such as MMP9, was quantified. These targets were selected based on their established relevance to cellular senescence within the context of bone loss as validated in prior studies conducted by our team.^6–9^ Detailed information regarding the Primer’s used in these experiments is provided in Table 1. For quantifying mRNA levels, the 2^−ΔΔCt^ method was employed to calculate the normalized RNA expression for each target gene relative to the control gene.

Table 1: List of Primers Used for RT-qPCR.

| **Genomic Target** | **Forward Primer Sequence** | **Reverse Primer Sequence** |
| --- | --- | --- |
| SOST | TCCTCCTGAGAACAACCA | CTGTACTCGGACACATCTT |
| MEPE | TGCTGCCCTCCTCAGAAATATC | GTTCGGCCCCAGTCACTAGA |
| *Cdkn2a (p16^Ink4a^)* | GAACTCTTTCGGTCGTACCC | AGTTCGAATCTGCACCGTAGT |
| *Cdkn1a (p21)* | GAACATCTCAGGGCCGAAAA | TGCGCTTGGAGTGATAGAAATC |
| *MMP9* | TGAGTCCGGCAGACAATCCT | CCCTGGATCTCAGCAATAGCA |
| GAPDH | ACTCAAGATTGTCAGCAAT | CCATCCACAGTCTTCTGGGT |
| L32 | CCATCTGTTTTACGGCATCATG | TGAACTTCTTGGTCCTCTTTTTGA |

**Immunofluorescence (IF) Staining:** To visualize the cytoskeleton during single-cell mechanical testing (i.e., nanoindentation), CellMask Deep Red Actin Tracking Stain (Thermo Fisher) was employed on live cells. Post-nanoindentation, cells underwent IF staining for F-actin and nuclei. The protocol involved washing the cells thrice with DPBS, fixing with 4% paraformaldehyde (PFA), and subsequent staining using CellMask for actin and DAPI for nuclei. Samples were imaged using our inverted fluorescence microscope on the Pavone system with 20x (NA=0.5) objective.


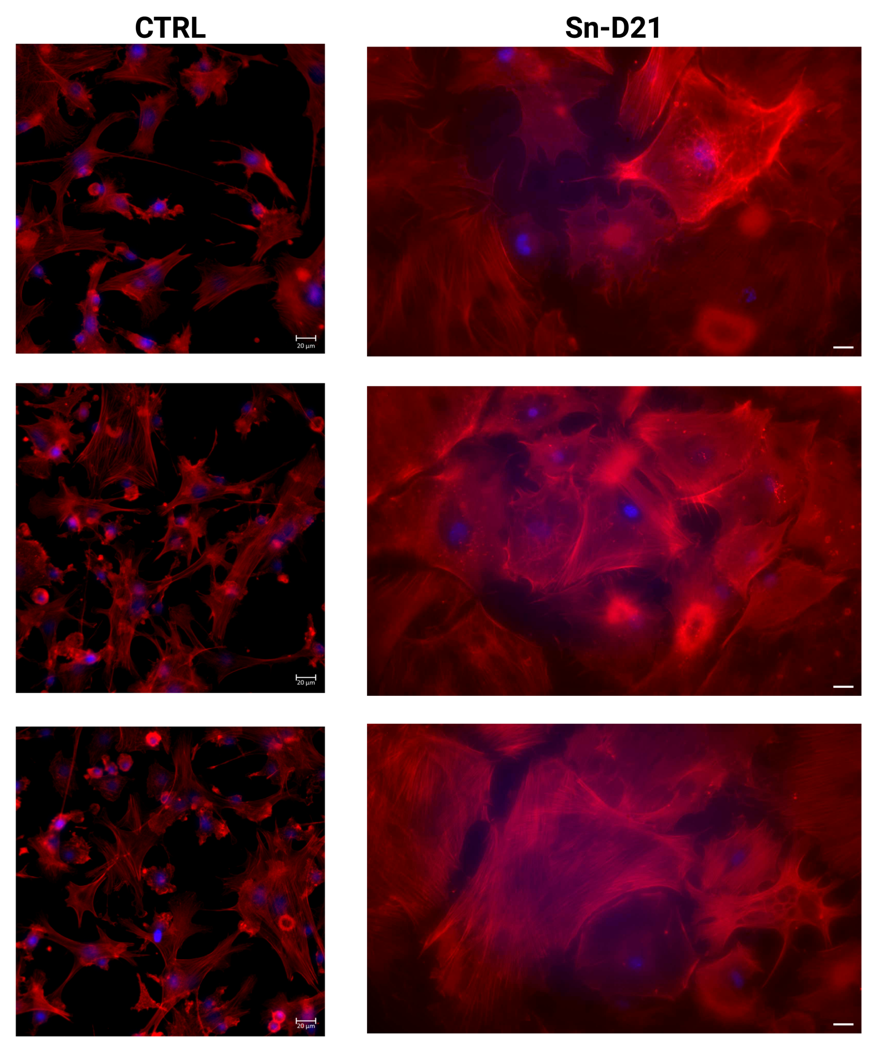


Figure S1: Representative IF micrographs (F-actin/DAPI) from different regions of CTRL and Sn-D21 culture conditions.

**Single cell mechanical testing:** Mechanical properties of the primary cells (2D culture) were measured at sub-cellular resolution using optical fiber-based interferometry nanoindenter (Pavone; Optics11Life). This instrument enabled single-cells mechanical characterization in live culture conditions. For these experiments, we used a spherical probe with R=3 µm and 0.019 N/m stiffness. The peak load threshold was set at 0.01 µN. A minimum of 30 indentation curves were obtained from each culture well at different locations (i.e., central and peripheral). We used Hertzian contact mechanics model^10–12^ with our load-indentation data to determine the Young’s modulus.

**Statistical analyses:** All statistical analyses were conducted using Prism9 software. We employed one-way and two-way ANOVA to analyze biophysical data (i.e., Young’s Modulus) and biomarker expressions, respectively, setting the significance level (α) at 0.05. Post-ANOVA, Tukey’s multiple comparison post hoc test was performed to determine significant differences among experimental groups, with results presented including 95% confidence intervals of the differences.

**References**

1. Farr JN, Fraser DG, Wang H, et al. Identification of Senescent Cells in the Bone Microenvironment. *Journal of Bone and Mineral Research*. 2016;31(11):1920-1929. doi:10.1002/jbmr.2892

2. Farr JN, Xu M, Weivoda MM, et al. Targeting cellular senescence prevents age-related bone loss in mice. *Nature Medicine 2017 23:9*. 2017;23(9):1072-1079. doi:10.1038/nm.4385

3. Farr JN, Saul D, Doolittle ML, et al. Local senolysis in aged mice only partially replicates the benefits of systemic senolysis. *J Clin Invest*. 2023;133(8):366-374. doi:10.1172/JCI162519

4. Stern AR, Stern MM, van Dyke ME, Jähn K, Prideaux M, Bonewald LF. Isolation and culture of primary osteocytes from the long bones of skeletally mature and aged mice. *Biotechniques*. 2012;52(6):361-373. doi:10.2144/0000113876

5. Lee BY, Han JA, Im JS, et al. Senescence-associated β-galactosidase is lysosomal β-galactosidase. *Aging Cell*. 2006;5(2):187-195. doi:10.1111/j.1474-9726.2006.00199.x

6. Chandra A, Lagnado AB, Farr JN, et al. Targeted Reduction of Senescent Cell Burden Alleviates Focal Radiotherapy-Related Bone Loss. *Journal of Bone and Mineral Research*. 2020;35(6):1119-1131. doi:10.1002/jbmr.3978

7. Farr JN, Fraser DG, Wang H, et al. Identification of Senescent Cells in the Bone Microenvironment. *Journal of Bone and Mineral Research*. 2016;31(11):1920-1929. doi:10.1002/jbmr.2892

8. Farr JN, Xu M, Weivoda MM, et al. Targeting cellular senescence prevents age-related bone loss in mice. *Nature Medicine 2017 23:9*. 2017;23(9):1072-1079. doi:10.1038/nm.4385

9. Farr JN, Saul D, Doolittle ML, et al. Local senolysis in aged mice only partially replicates the benefits of systemic senolysis. *J Clin Invest*. 2023;133(8):366-374. doi:10.1172/JCI162519

10. Wu G, Gotthardt M, Gollasch M. Assessment of nanoindentation in stiffness measurement of soft biomaterials: kidney, liver, spleen and uterus. *Scientific Reports 2020 10:1*. 2020;10(1):1-11. doi:10.1038/s41598-020-75738-7

11. Lee W, Ostadi Moghaddam A, Shen S, et al. An optomechanogram for assessment of the structural and mechanical properties of tissues. *Scientific Reports 2021 11:1*. 2021;11(1):1-12. doi:10.1038/s41598-020-79602-6

12. Qian L, Zhao H. Nanoindentation of Soft Biological Materials. *Micromachines 2018, Vol 9, Page 654*. 2018;9(12):654. doi:10.3390/MI9120654
